# Comparative analysis of the *JRL* gene family in the whole-genome of five gramineous plants

**DOI:** 10.3389/fpls.2024.1501975

**Published:** 2024-12-24

**Authors:** Luping Gong, Yitong Lu, Yujie Wang, Furu He, Tao Zhu, Baoping Xue

**Affiliations:** ^1^ College of Life Science and Engineering, Henan University of Urban Construction, Pingdingshan, China; ^2^ Department of Plant Sciences, College of Life Sciences, Wuhan University, Wuhan, China

**Keywords:** *JRL* gene family, evolution, expression pattern, stress response, gramineous plants

## Abstract

The Jacalin-related lectins (*JRLs*) gene family play a crucial role in regulating plant development and responding to environmental stress. However, a systematic bioinformatics analysis of the *JRL* gene family in Gramineae plants has been lacking. In this study, we identified 101 JRL proteins from five Gramineae species and classified them into eight distinct clades. Most of the AtJRL proteins clustered in the same group and were differentiated from the Gramineae JRL proteins. The analysis of protein motifs, gene structures and protein domain revealed that the *JRL* genes play diverse functions in Gramineae plants. Duplication events indicated that tandem duplication significantly contributed to the expansion of the JRL family, with most JRL members underwent purifying selection. Tissue expression profile analysis showed that most *OsJRL* genes were highly expressed in the roots, while *ZmJRL* genes exhibited high expression in inflorescences. Furthermore, the expression level of *OsJRL* and *ZmJRL* genes were influenced by drought, cold, heat and salt stresses, respectively, implying that these genes play important roles in response to various abiotic stresses. RT-qPCR results demonstrated that *OsJRL4* was up-regulated under PEG6000 and NaCl stresses, while *OsJRL12* and *OsJRL26* were down-regulated under PEG6000. These findings provide comprehensive insights into the *JRL* gene family in Gramineae plants and will facilitate further functional characterization of JRLs.

## 1 Introduction

The Poaceae family comprises the most widely distributed monocotyledonous plants, and is also the most economically significant large family. Gramineous plants serve as the primary source of human food and livestock feed, functioning as essential raw materials for the production of starch, sugar, wine, paper, textiles, and construction materials. In recent years, the rapid advancement of genomics has led to the sequencing for an increasing number of Gramineous plant genomes (Mockler et al., 2010; Tao et al., 2021; Shang et al., 2023; He et al., 2024). The successful completion of whole-genome sequencing for these species established a robust foundation for further exploration of genomic information, evolutionary conduction and genetic studies. The genomes of Gramineous plants have primarily evolved through a process of gene duplication and loss (Shuai et al., 2002; Lin et al., 2003). Following these events, duplicated genes may experience either purifying or positive selection thus facilitating adaptive evolution. Although these genomes exhibit certain degrees of collinearity, the genomic information varies significantly among species. Notably, many gene families within Gramineae plants display species-specific characteristics due to gene duplication or loss across different species (Gossmann et al., 2010; Duan et al., 2023; Liu et al., 2024).

Plant lectins, exhibits unique molecular structure, are being classified into three types of ‘merolectins’, ‘hololectins’, and ‘chimerolectins’ (Tsaneva and Van Damme, 2020). JRL proteins, known as new plant lectins, play crucial roles in mediating stress signaling pathways across various plant species (Subramanyam et al., 2008; De Coninck and Van Damme, 2021; Marothia et al., 2023). Early studies have highlighted JRL isoforms, *Orysata1* and *Orysata2*, in responding to NaCl stress (Zhang et al., 2000). Recent studies indicate that the *PeDJ01* from moso bamboo is up-regulated in response to salt or cold stress, highlighting its significant role in stress regulation (Ma et al., 2021). Over-expression of *OsJAC1* in rice enhances resistance to *oomycetes* and fungi, with its JRL domain being crucial for binding to penetration site of *powdery mildew* (Weidenbach et al., 2016). Furthermore, OsJAC1 and wheat VER2 play a important role in lignan formation (Burlat et al., 2001; Jiang et al., 2006; Wang and Ma, 2005; Yong et al., 2003).

The expression levels of *JRL* genes are responsive to various plant hormones. Notably, *OsJAC* demonstrates high transcription levels in leaves and is inducible by jasmonate (JA) treatment (Jiang et al., 2006). The JRL-like protein TaJRLL1, which contains two jacalin-like lectin domains, is involved in the salicylic acid (SA)/JA signaling pathway (Xiang et al., 2011). In *Helianthus tuberosus* callus, the level of *HTA* gene were notably up-regulated by MeJA treatment (Nakagawa et al., 2000). Although, the expression of *LEM2* was rapidly induced by SA, it was decreased when responding to drought, dehydration and abscisic acid (ABA) treatments (Abebe et al., 2005).

The sequencing of plant genomes has enabled the identification of *JRL* genes in *Arabidopsis*, rice, and wheat (Nagano et al., 2008; Jiang et al., 2010; Song et al., 2013). However, comprehensive bioinformatic analysis of *JRL* genes in Gramineous plants is lacking. In this study, we screened and identified JRL family members in the genomes of 5 Gramineae plants and examined their evolutionary relationships, cis-acting elements, gene collinearity, and duplication events. RNA-seq and RT-qPCR results were utilized to explore the expression patterns of *JRL* genes under diverse abiotic stresses. This research serves as a valuable resource for further researches on the biological functions and stress resistance mechanisms involving *JRL* genes in plants.

## 2 Results

### 2.1 Identification and phylogenetic analysis of JRL family members

**Figure 1:**
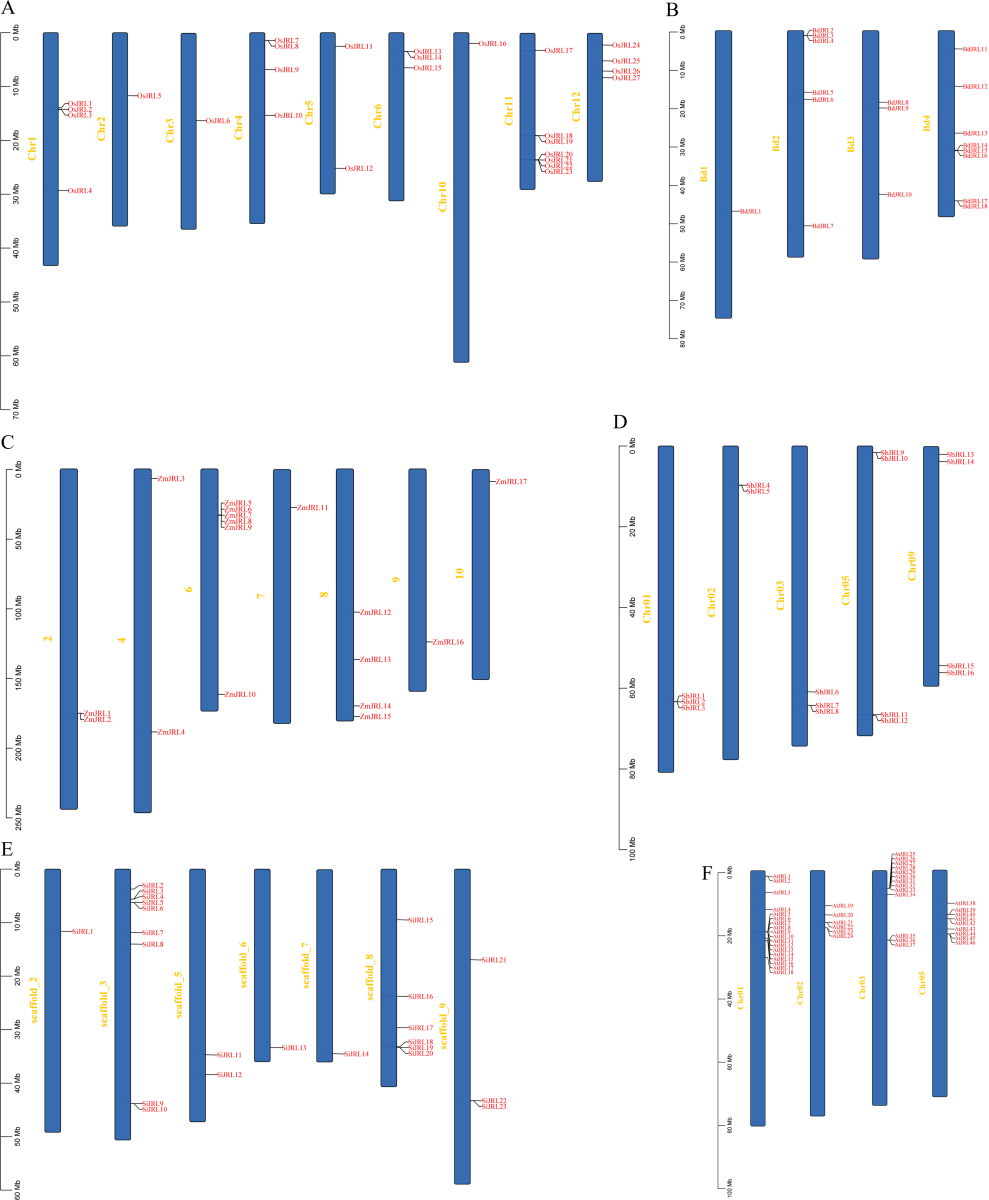
The chromosomal location of the *JRL* genes in six species, including **(A)** *O. sativa*, **(B)** *B*. *distachyon*, **(C)** *Z. mays*, **(D)** *S. bicolor*, **(E)** *S. italica* and **(F)** *A*. *thaliana*.

In this study, 27, 18, 17, 16, and 23 JRL protein members were identified from *Oryza sativa* (*O. sativa*), *Brachypodium distachyon* (*B. distachyon*), *Zea mays* (*Z. mays*), *Sorghum bicolor* (*S. bicolor*), and *Setaria italica* (*S. italica*), respectively (**Figure 1**; **Supplementary Table 1**). We found that *O. sativa* has more *JRL* genes than *S. italica*, *S. bicolor*, *B. distachyon* and *Z. mays*, implying a gene expansion of the *JRL* gene family among different Gramineae species. Further, we named these *JRL* genes according to their location on the chromosomes: *OsJRL1*-*OsJRL27*; *BdJRL1*-*BdJRL18*; *ZmJRL1*-*ZmJRL17*; *SbJRL1*-*SbJRL16* and *SiJRL1*-*SiJRL23* (**Figure 1**). The *JRL* genes of *O. sativa* are distributed on 9 chromosomes (**Figure 1A**). *BdJRL* genes (**Figure 1B**) and *AtJRL* genes (**Figure 1F**) are focusly distributed on 4 chromosomes. While *ZmJRL* genes and *SiJRL* genes are distributed on 7 chromosomes, respevtively (**Figures 1C, E**), *SbJRL* genes are distributed on chromosomes 1, 2, 3, 5, and 9 (**Figure 1D**). And these *JRL* genes are often distributed together in clusters (**Figure 1**).

**Figure 2:**
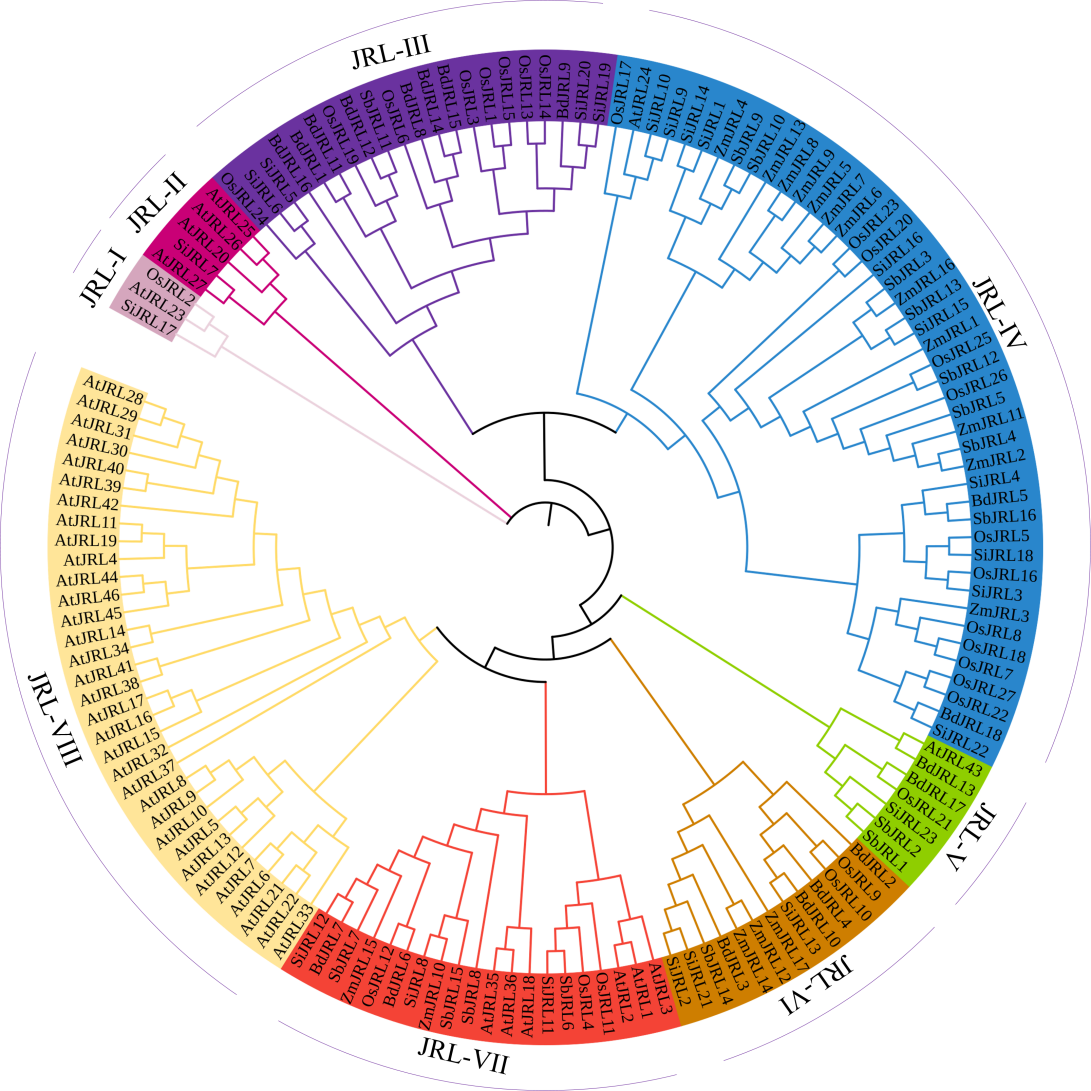
The evolutionary analysis of the 147 identified JRLs proteins sequences from *O. sativa*, *Z. mays*, *B.distachyon*, *S. bicolor*, *S. italica*, and *Arabidopsis*. 147 JRLs were divided into eight subgroups of JRL-I, JRL-II, JRL-III, JRL-IV, JRL-V,JRL-VI, JRL-VII and JRL-VIII respectively. The evolutionary tree was analyzed by MEGA 7 (method: neighbor-joining (N-J); bootstrap values: 1000 replicates).

To investigate the evolutionary mechanism of JRL proteins in Gramineous, we used the Neighbor-Joining (N-J) method to construct an evolutionary tree including 46 AtJRL, 27 OsJRL, 23 SiJRL, 16 SbJRL, 18 BdJRL and 17 ZmJRL proteins. As shown in **Figure 2**, these JRL proteins are divided into 8 subgroups. The JRL-IV group contains the most members, including 12 OsJRL, 1 AtJRL, 10 SiJRL, 12 ZmJRL, 8 SbJRL, and 2 BdJRL members. In contrast, JRL-I contains the fewest JRL members. Interestingly, the JRL-III and JRL-VI groups only include the Gramineous JRL proteins. And JRL-VIII is unique to *Arabidopsis*, containing 33 AtJRL proteins (71.7%). In addition, we found that the ZmJRL is lost in the group of JRL-III and JRL-V. Meanwhile, both JRL-IV and JRL-VII contain members of Gramineae JRL and *Arabidopsis* JRL, suggesting that JRL members in these two groups may function conserved between *Arabidopsis* and Gramineae (**Figure 2**).

### 2.2 Gene structure and protein motif analysis

**Figure 3:**
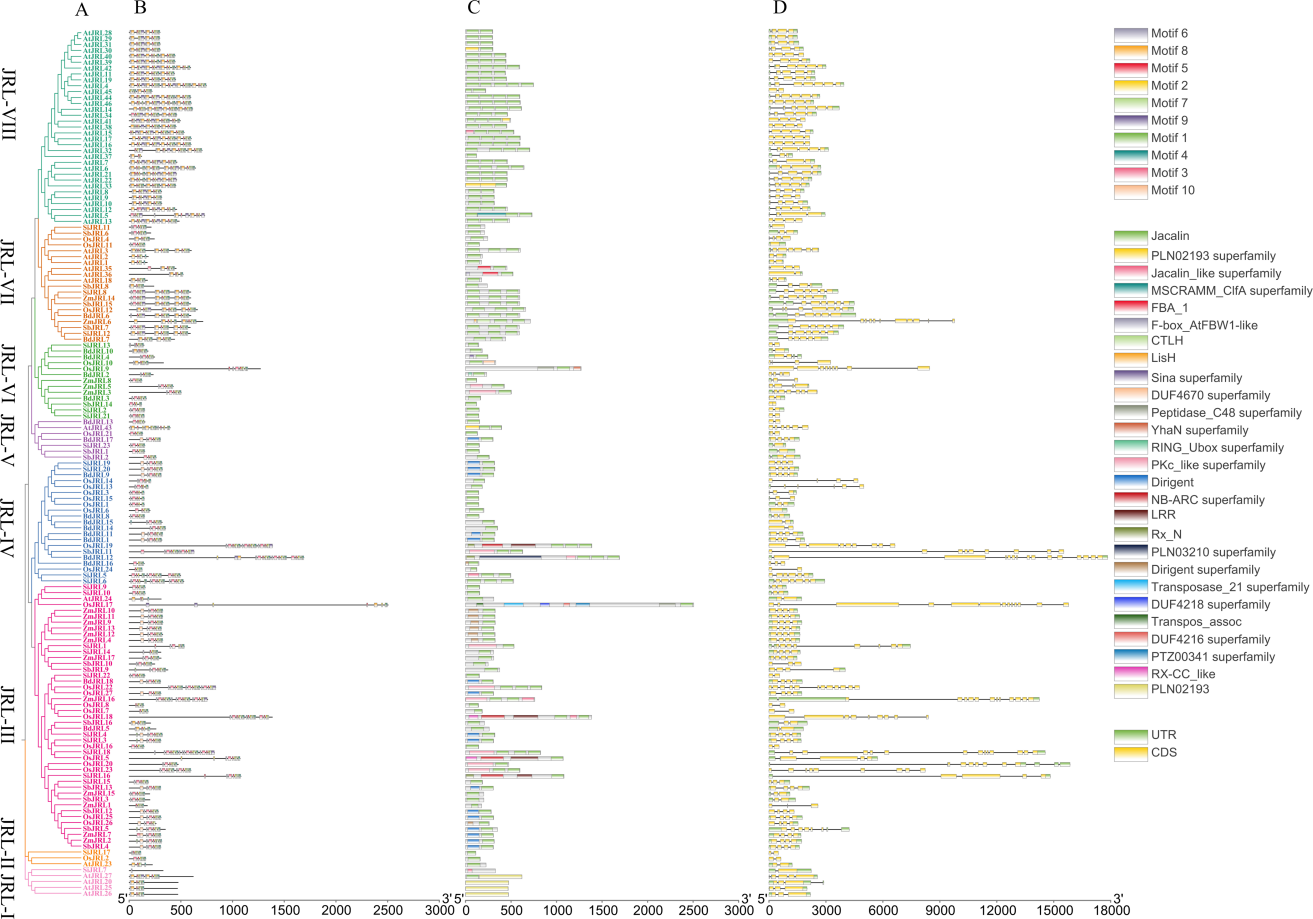
Phylogenetic relationship, conserved motif, conserved domain and gene structure analysis of JRLs in Gramineae and *Arabidopsis*. **(A)** Phylogenetic tree of JRL proteins in Gramineae and *Arabidopsis*. The phylogenetic tree was constructed with MEGA 7 through neighbor-joining method using the full-length amino acid sequences of JRL proteins, and the bootstrap test replicate was set as 1000. **(B)** Distributions of conserved motifs in JRL proteins in Gramineae and *Arabidopsis*. Ten putative motifs are indicated in different colored boxes. **(C)** The conserved domain of JRL full-length amino acid sequences in Gramineae and *Arabidopsis*. **(D)** Exon/intron organization of *JRL* genes in Gramineae and *Arabidopsis*. Yellow boxes represent exons, gray lines represent introns and green boxes represent UTR.

The diversity of gene structure can reflect the evolutionary processes of gene family. We analyzed the *JRL* gene structure, protein motif and conserved domain in *O. sativa*, *B. distachyon*, *Z. mays*, *S. bicolor*, *S. italica* and *A. thaliana*. The protein motif analysis showed that nearly all JRL proteins contain motif 3, motif 4, motif 8, motif 9 and motif 10, indicating that these motifs may be conserved for the functions roles of JRL proteins. We observed that JRL members of the same subgroup exhibit similar conserved motifs (**Figures 3A, B**). In contrast to the other six subgroups, members of the JRL-VII and JRL-VIII subgroups contain two or three Motif 1 and Motif 2 in tandem. While JRL-VIII comprises only *Arabidopsis* members, JRL-VII includes both Gramineae *JRL* genes and *AtJRL* genes. These suggests that the members of these two subgroups may possess unique functions in the evolutionary process, warranting further investigation.

We found that AtJRL28, AtJRL29, SiJRL5, and BdJRL7 contain two “Jacalin domain”, while some JRL proteins like SiJRL8, ZmJRL14, OsJRL12, and SbJRL15 possess three “Jacalin domain”. Interestingly, we found the members in JRL-VII and JRL-VIII subgroups contain 1-5 Jacalin domains. However, the conserved domain of *S. bicolor* is relatively simple, comprising only three types of domains: Jacalin, Dirigent and PKc_like superfamily. In contrast, the OsJRL members contain more complex conserved domains (**Figures 3A, C**). These suggests that there are both functional conservation and functional differentiation among the Gramineae JRL family members. Moreover, most AtJRL proteins that only contained Jacalin domain and no other domains (**Figure 3**). Notably, AtJRL proteins are more likely to have multiple tandem Jacalin domain repeats. For example, AtJRL4 is composed of 5 tandem Jacalin domains (**Figures 3A, C**). It is noteworthy that a dirigent domain, which is the key domain of DIR proteins (Gong et al., 2023), was found at the N-terminal of the Gramineous JRL proteins, but was not present in *Arabidopsis*.

The number of exons in *JRL* genes ranged from 2 to 14 (**Figures 3A, C, D**). In *O. sativa*, the number of introns varied from 1 to 12. However, *AtJRL* genes exhibited a narrower intron range of 1-6 (**Figure 3D**). Furthermore, we observed that members of the same subgroup tend to exhibit similar gene structures. Previous studies have indicated that in the same gene family, genes with multiple introns play significant roles in stress resistance compared to those without introns (Shaul, 2017). The considerable variation in gene structure among Gramineous plants suggests potential substantial differences in gene function.

### 2.3 Analysis of duplication event of *JRL* genes

**Figure 4:**
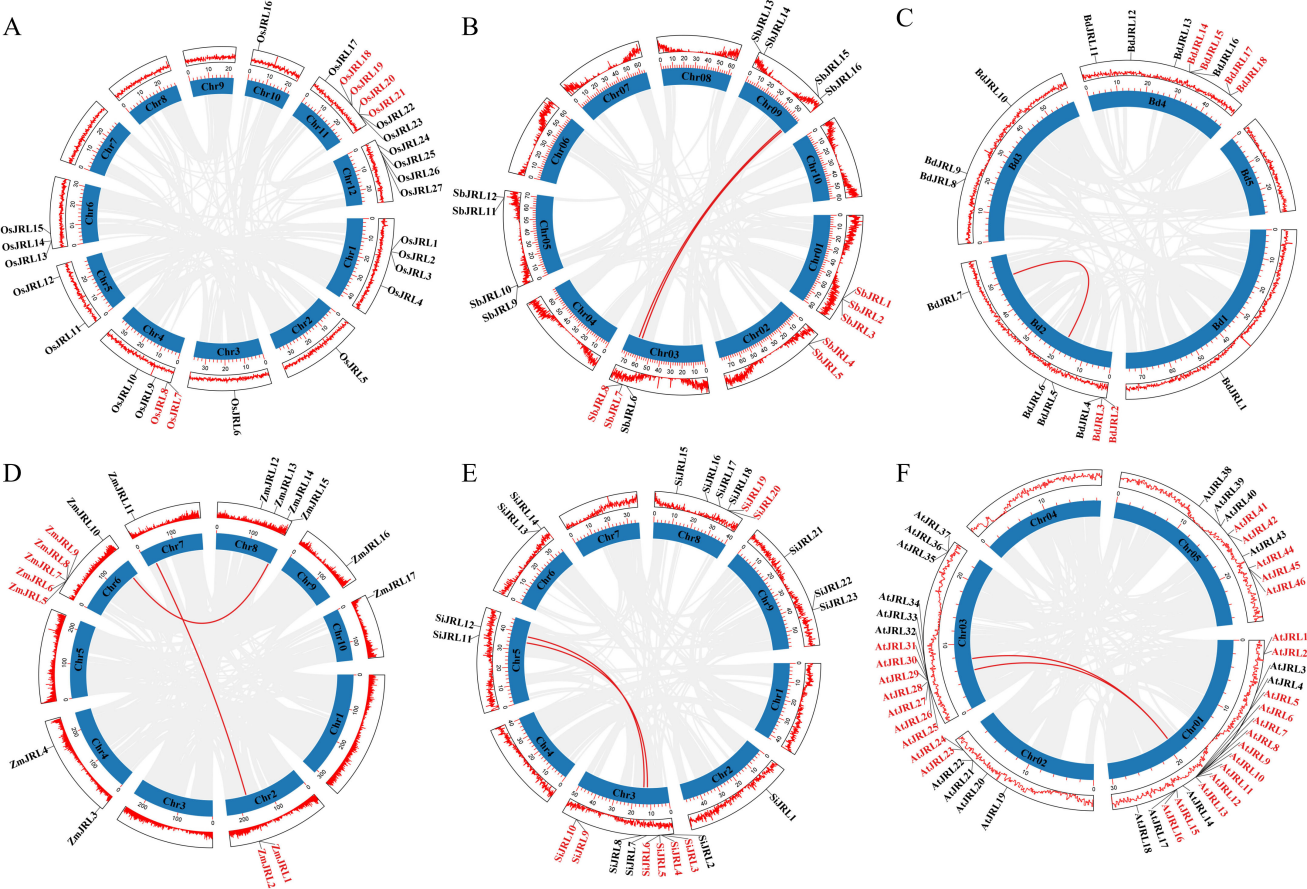
The duplication events analysis of *JRL* genes in Gramineae and *Arabidopsis*, including **(A)** *O. sativa*, **(B)** *S. bicolor*, **(C)** *B*. *distachyon*, **(D)** *Z. mays*, **(E)** *S. italica* and **(F)** *Arabidopsis.* The name of TD genes are indicated in red color. The WGD genes are indicated in red lines.

During evolution, tandem duplication (TD) and whole genome duplication (WGD) have played crucial roles in the expansion of gene families. In this study, we analyzed the duplication events of the *JRL* gene family members in *O. sativa*, *Z. mays*, *S. italica*, *S. bicolor*, *B. distachyon* and *Arabidopsis*. As shown in **Figure 4**; **Supplementary Table 2**, we identified 61 TD and 18 WGD genes. Specifically, we found 6, 7, 6, 7, 8, and 27 TD genes, and 0, 4, 2, 4, 4 and 3 WGD genes in *O. sativa*, *S. bicolor*, *B. distachyon*, *Z. mays*, *S. italica*, and *Arabidopsis*, respectively. The TD genes of *AtJRL6-13* (**Figure 4**) are predominantly located in Group VIII of **Figure 2**. Moreover, some TD genes of *O. sativa*, *Z. mays*, *S. italica*, *S. bicolor* and *B. distachyon* were grouped into the same subfamily (**Figure 2**), suggesting that the expansion of *JRL* gene families in *Z. mays*, *S. italica*, *S. bicolor* and *B. distachyon* is primarily driven by the significant expansion of a specific subfamily. It can be inferred that both TD and WGD events jointly promote the expansion of *JRL* gene families in *O. sativa*, *Z. mays*, *S. italica*, *S. bicolor*, *B. distachyon* and *Arabidopsis*, with TD playing a a major dominant role.

**Figure 5:**
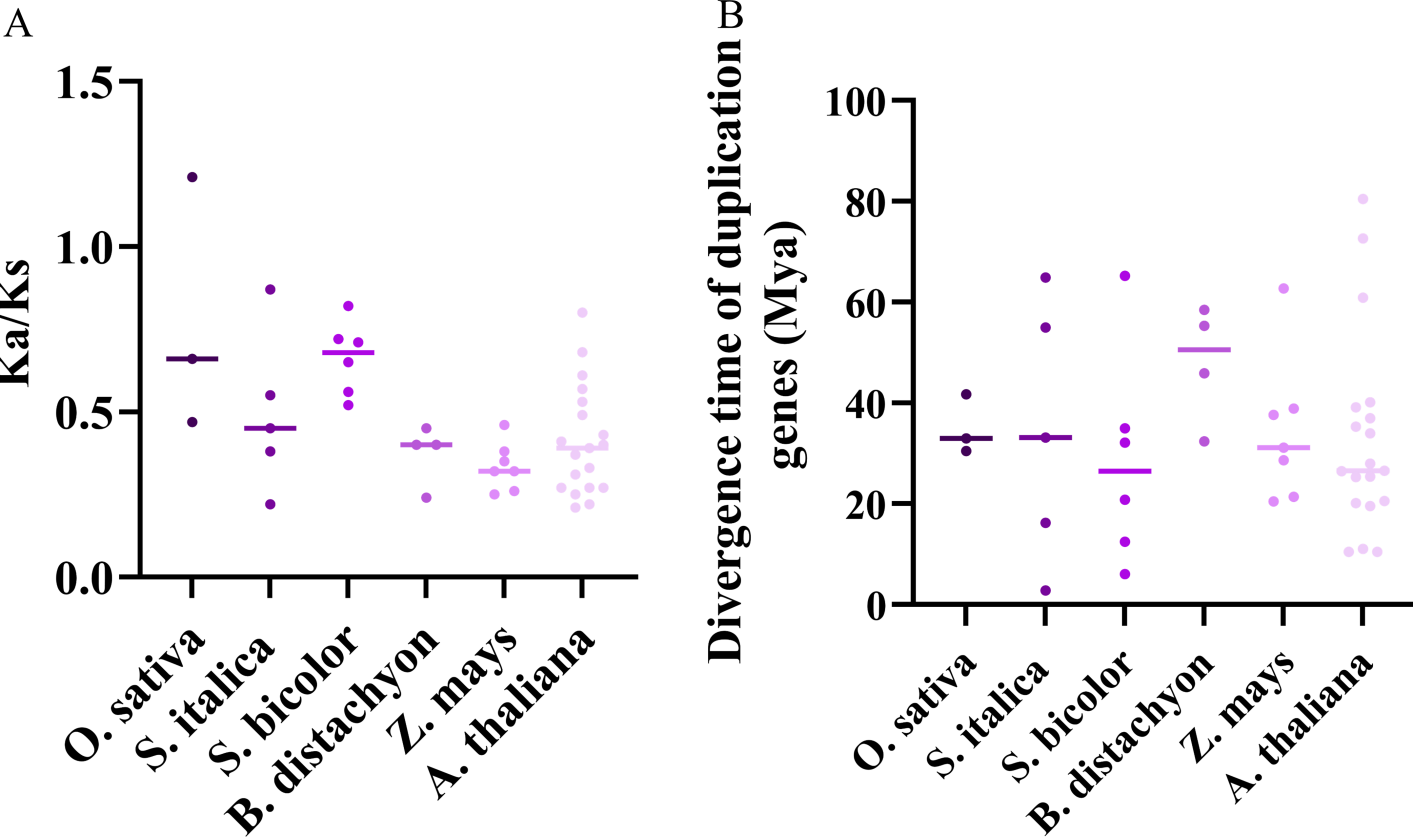
Selection pressure and divergence time analysis of *JRL* gene pairs in *O. sativa*, *A*. *thaliana*, *S. italica*, *B.distachyon*, *S.bicolor* and *Z. may*. **(A)** Ka/Ks ratio calculation of the *JRL* gene pairs. **(B)** The divergence time prediction of *JRL* gene pairs.

Further, we calculated the ratio of Ka/Ks on TD and WGD gene pairs. And except for one gene pair in rice was larger than 1, the ratio of Ka/Ks of all other replicated gene pairs were smaller than 1 (**Figure 5A**). Additionally, we evaluated the appearance time of these TD and WGD genes. And the divergence time of TD genes in the Z*mJRL*, *BdJRL*, *SiJRL*, *OsJRL*, *SbJRL* and *AtJRL* ranged from 2.77 to 62.68, while WGD genes in these species ranged from 31.15 to 80.44 (**Figure 5B**). These results hint that most duplicate genes of the *JRL* family were under purification selection, and TD duplication events might contributed chiefly to their evolution in *O. sativa*, *A. thaliana*, *S. italica, B.distachyon*, *S.bicolor* and *Z. may.*

### 2.4 Collinearity analysis

**Figure 6:**
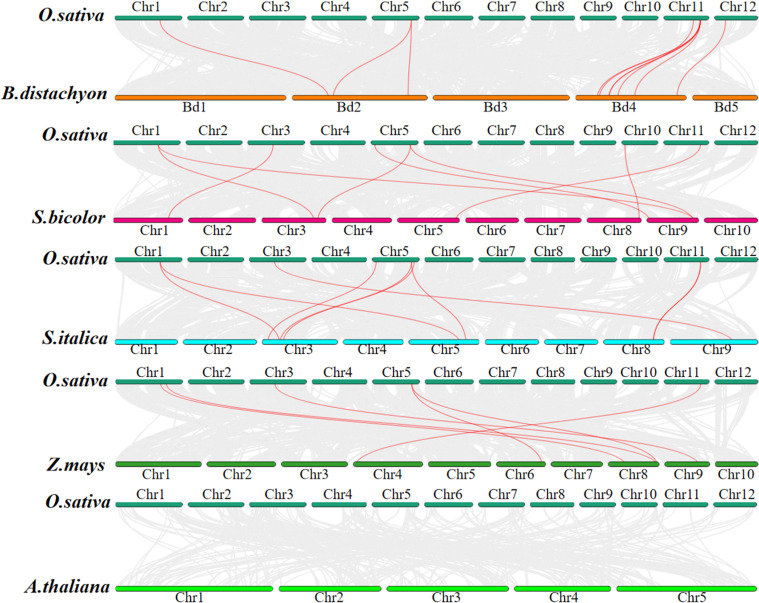
Synteny analysis of *O. sativa* between and *B*. *distachyon*, *S. bicolor*, *S. italica*, *Z. mays* and *A*. *thaliana*, respectively. The collinear blocks between *O.sativa* and other plants were shown in gray lines. The syntenic *JRL* gene pairs between *O.sativa* and other plants were highlighted in red lines.

To further investigate the evolutionary mechanism of the *JRL* gene, we conducted a collinearity analysis across *O. sativa*, *B. distachyon*, *S. bicolor*, *S. italica*, *Z. mays*, and *A. thaliana*. As shown in **Figure 6**, the number of collinear gene pairs between *O. sativa* and the other species are follows: 9 with *B. distachyon*, 8 with *S.bicolor*, 8 with *S. italica*, 6 with *Z. may* and 0 with *A. thaliana*. It is noteworthy that some *OsJRL* genes exhibit collinear relationships with at least two genes. For example, *OsJRL4* is collinear with *SbJRL6* and *SbJRL16*; *OsJRL12* is collinear with *SbJRL7*, *SbJRL15*, *ZmJRL10* and *ZmJRL15*.. These findings suggest that these genes may represent significant genetic resources. Notably, the genetic relationship between *O. sativa* and *B. distachyon* is closer than that observed among *S. italica*, *Z. may*, and *S.bicolor*. However, no co-linear gene pairs between *O. sativa* and *A. thaliana* was found, which demonstrates the function differences between monocotyledonous and dicotyledonous plants, to some extent.

### 2.5 Analysis of promoter elements

**Figure 7:**
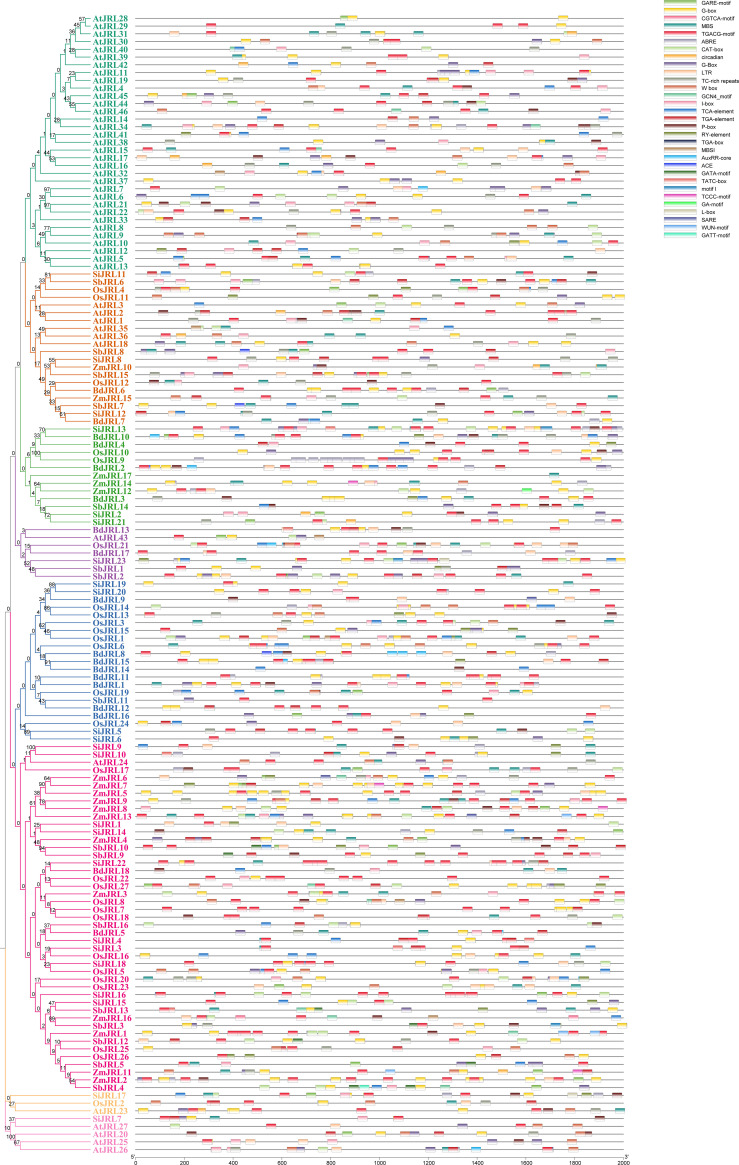
Distribution of cis-acting elements of *JRL* gene family. The cis-elements were predicted in the 2000 bp promoter sequences of *JRL* genes. The Cis-elements was analyzed by plantcare.

To better understand the function, we analyzed the cis-elements of 147 *JRL* genes using their 2k-length promoter sequences. Our results revealed a strong connection between the *JRL* gene and various processes, such as meristem expression, circadian rhythm, root-specific and seed-specific regulatory elements. In addition, the *JRL* gene displayed a wide array of responsiveness to plant hormones, indicating its involvement in a complex hormone signaling network. And the most prevalent observed elements included abscisic acid response elements (ABRE) followed by jasmonic acid response elements (CGTCA-motif and TGACG-motif), implying the significant role of the *JRL* gene in coping with abiotic stress. The existence of light-responsive elements such as G-box and I-box implies that *JRL* genes might play a role in photosynthesis. The discovery of transcription factor binding sites, including W-box, indicates that multiple transcription factors may regulate the *JRL* genes’ transcription. Additionally, we observed that the cis-elements of MBSI associated with flavonoid biosynthesis was found in *A. thaliana, O.sativa*, and *Z. mays*, but not in *B. distachyon, S. italica* and *S. bicolor*. On the contrary, the cis-elements of motif I associated with root specific was found in *S. italica* and *S. bicolor*, but not in other four species (**Figure 7**; **Supplementary Table 3**). Moreover, members of the same subgroup have similar cis-acting elements (**Figure 7**). These results indicate that the *JRL* genes may simultaneously have functional consistency and differences.

### 2.6 Tissues expression pattern analysis of *OsJRL* and *ZmJRL* genes

**Figure 8:**
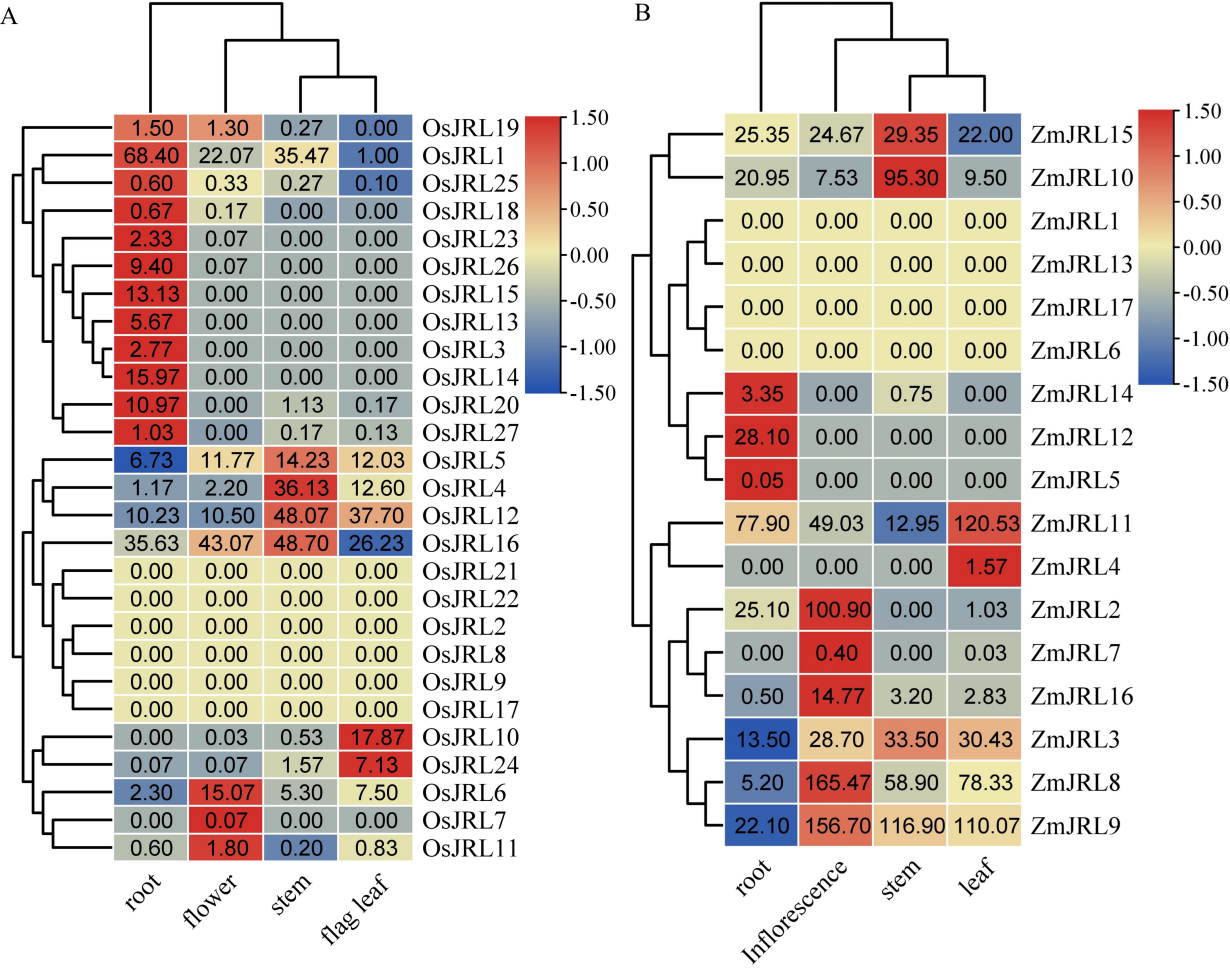
Tissue expression pattern analysis of *OsJRL* and *ZmJRL* genes in different tissues. **(A)**. The expression heat map of *OsJRL genes* in 4 tissues (root, flower, stem, and flag leaf). **(B)**. The expression heat map of *ZmJRL genes* in 4 tissues (root, inflorescence, stem, and leaf). The data are gene expression that caculated by row-scaled FPKM values, and the heatmap is constructed by TBtools software. Red and blue boxes indicate high and low expression levels of *OsJRL* and *ZmJRL* genes, respectively.

To enhance our understanding about the potential function of the *JRL* genes, we analyzed the tissue expression patterns of *OsJRL* and *ZmJRL* genes. Our findings revealed that *OsJRL2/8/9/17/21/22* and *ZmJRL1/6/13*/*17* were not expressed in any of the four examined tissues (**Figure 8**; **Supplementary Table 4**). And 44.44% of *OsJRL* genes and 17.65% of *ZmJRL* genes exhibited high expression levels in roots, while 11.11% of *OsJRL* genes and 29.41% of *ZmJRL* genes were highly expressed in flowers. *OsJRL3/13/14/15/23/26* and *ZmJRL5/12* were exclusively expressed in roots, whereas *OsJRL7* and *ZmJRL7* were exclusively expressed in flowers. Additionally, *OsJRL4/5/12/16* and *ZmJRL10/15* were highly expressed in stems; *OsJRL10/24* and *ZmJRL4/11* are highly expressed in leaf (**Figure 8**). Interestingly, the collinear gene pairs *OsJRL6/ZmJRL16* were both highly expressed in their inflorescence. And the collinear genes *OsJRL12*, *ZmJRL10* and *ZmJRL15* demonstrated high expression levels in stems (**Figure 8**). However, the tissue expression patterns of the collinear gene pairs *OsJRL20/ZmJRL3* exhibited differently: *OsJRL20* was highly expressed in roots, while *ZmJRL3* showed broad expression across root, flower, stem, and leaf.

### 2.7 Expression pattern analysis under abiotic stress treatments

**Figure 9:**
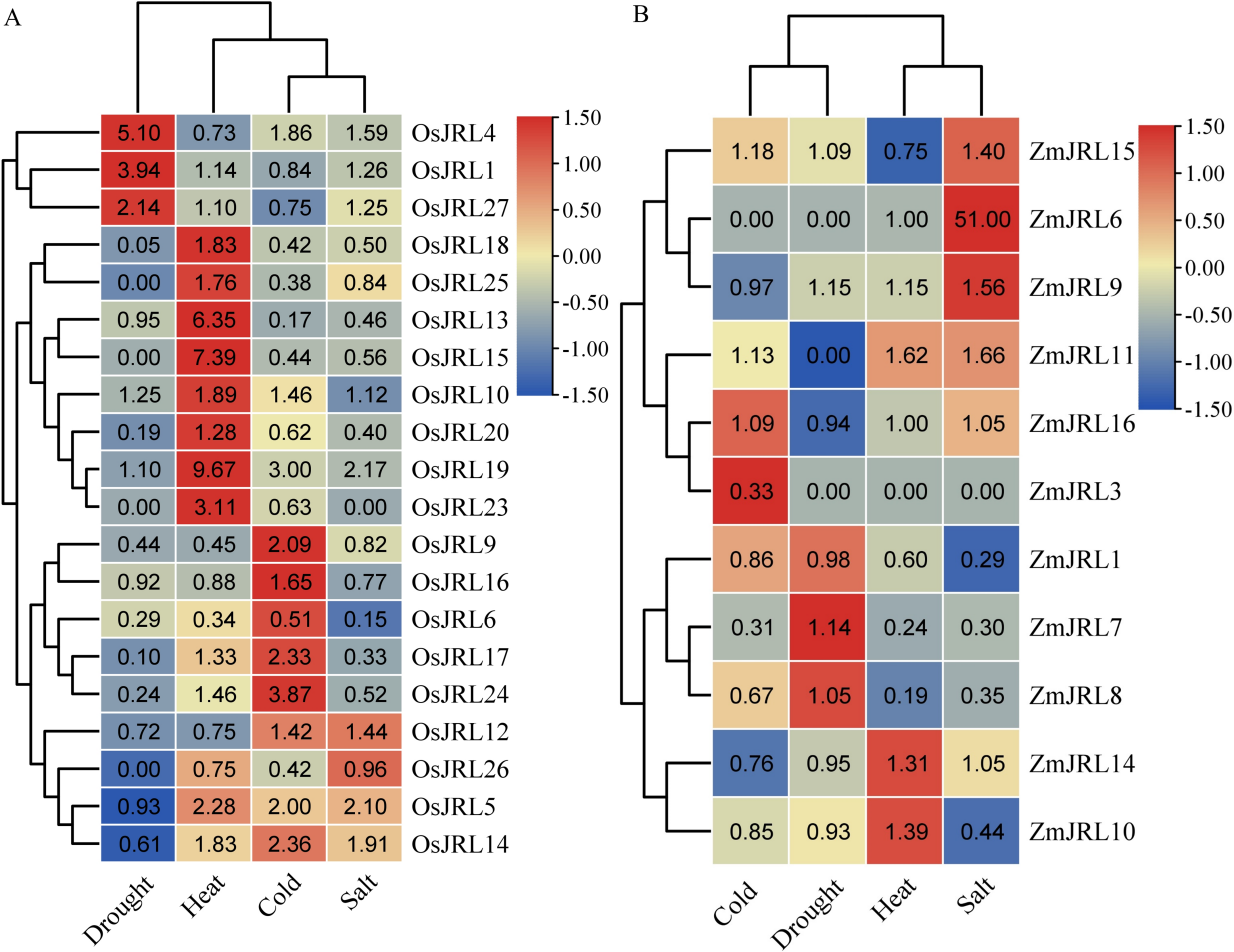
Expression profiles of the *OsJRL* and *ZmJRL* genes. **(A)** The heat map exhibit the ratio of the expression levels of *OsJRL* genes under abiotic stresses treatment (drought, heat, cold and salt). **(B)** The heat map exhibit the ratio of the expression levels of *ZmJRL* genes under abiotic stresses treatment (cold drought, heat, and salt). The data are gene expression that caculated by row-scaled FPKM values, and the heatmap is constructed by TBtools software. Red and blue boxes indicate high and low expression levels of *OsJRL* and *ZmJRL* genes, respectively.

For further investigation, we explored the expression levels of *ZmJRL*s and *OsJRLs* under abiotic stress by mining transcriptome data. As shown in **Figure 9**; **Supplementary Table 5**, the expression levels of *OsJRL1/4/27* genes were significantly up-regulated, while *OsJRL6/9/17/18/20/24* genes were down-regulated under drought stress. Under salt, heat and cold stresses, *OsJRL5/14* genes showed similar up-regulated expression patterns. Interestingly, some *OsJRL* genes displayed opposing expression patterns under different stress treatments. For instance, the expression levels of *OsJRL15/18/20/23* were up-regulated under heat stress, but down-regulated under drought, salt and cold stresses (**Figure 9A**). In addition, we observed that the expression levels of *ZmJRL6/9/11/15* genes were up-regulated under salt stress, with *ZmJRL6* showing an extremely significant up-regulation to approximately 51-fold. The expression level of the *ZmJRL3* gene was slightly up-regulated under cold stress, but it did not respond to drought, salt and heat stress (**Figure 9B**). Moreover, we note a descending trend in the expression levels of *ZmJRL1* and *ZmJRl10* under salt stress.

**Figure 10:**
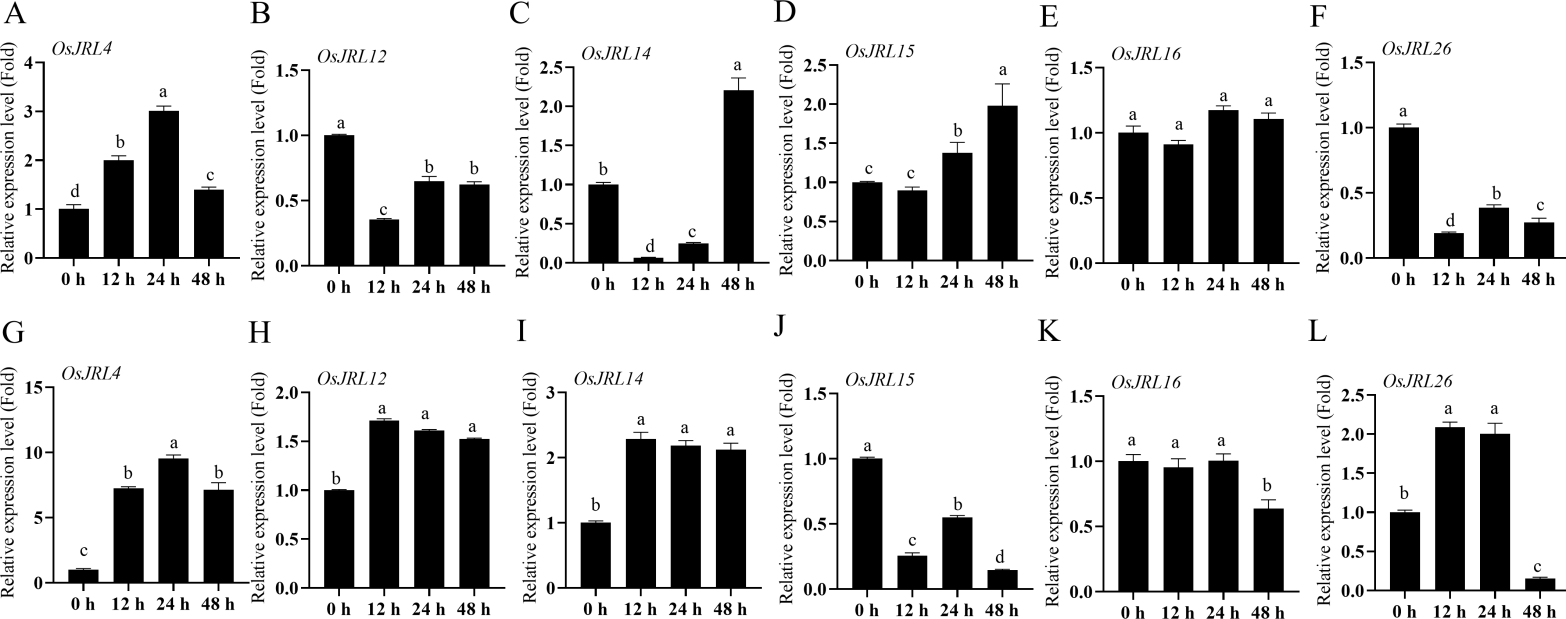
Expression patterns of *OsJRL* genes under abiotic stress by RT-qPCR. **(A–F)** The expression level of *OsJRLs* gene under 30% PEG6000 treatment. **(G-L)** The expression level of *OsJRLs* under 150 mM NaCl treatment. The expression levels were calculated using 2^-△△Ct^ methods. Means ± SDs (n = 3). The expression of 0 h were regarded as “1”. Different lowercase letters indicate significant differences among means (*P*<0.01). qRT-PCR analysis were performed using the rice *OsActin* as an internal control.

To further explore the potential function of the *JRL* gene, we randomly selected six *OsJRL* genes that were expressed in roots and responsive to abiotic stress. The mRNA levels of these six genes were assessed using RT-qPCR following treatment with 30% PEG6000 and 150 mM NaCl, respectively (**Figure 10**). The transcription level of *OsJRL4* was significantly up-regulated under PEG6000 treatment and peaking at 24 h, which was similar to its response under salt stress (**Figures 10A, G**). The expression levels of *OsJRL12* and *OsJRL26* were down-regulated under 30% PEG6000 treatment (**Figures 10B, F**), while *OsJRL15* exhibited a significant up-regulation (**Figure 10D**). Under salt stress, the expression levels of *OsJRL12*, *OsJRL14* and *OsJRL2*6 were significantly up-regulated (**Figures 10H, I, L**), whereas *OsJRL15* was inhibited (**Figure 10J**). Interestingly, the expression level of *OsJRL26* was initially increased and then decreased under salt stress (**Figure 10L**).

## 3 Discussion

In this study, a comprehensive analysis of 101 JRL proteins from five Gramineous plants have been carried out. And we then focused on rice and maize to investigate their expression patterns in different tissues, as well as their expression characteristics in response to various stresses.

Here, we identified 27, 17, 23, 16, and 18 *JRL* genes from *O. sativa*, *Z. mays*, *S. italica*, *S. bicolor* and *B. distachyon*, respectively (**Figure 1**; **Supplementary Table 1**). With the exception of *O. sativa* and *S. italica*, the number of *JRL* gene in the Gramineous family is relatively similar. Gramineous plants diverged approximately 50-70 million years ago (Paterson et al., 2003) and may have expanded at a comparable rate. Monocotyledonous and dicotyledonous plants diverged around 140 to 150 million years ago (Kumar et al., 2017). And the number of *JRL* genes in *Arabidopsis* (46) (Nagano et al., 2008) is approximately twice that of Gramineous plants, indicating a faster expansion rate of *JRL* genes in *Arabidopsis*.

The domains of JRL proteins exhibit considerable variability, with significant differences observed between Gramineous species and *Arabidopsis*. Gramineous JRL proteins can contain up to 3 Jacalin domains, while AtJRLs may possess as many as 5 tandem Jacalin domains (**Figure 3**), indicating that only one complete domain is required for the normal functioning of JRL proteins. And the formation of multiple domains may be necessary for the conformation of a specific protein structure, or it may be necessary for plants to adapt to complex environmental changes. Also, these differences in JRL protein domains may suggest that monocotyledonous and dicotyledonous plants have evolved distinct strategies. Some JRL protein domains are connected to the dirigent domains (**Figure 3**), which may be related to their specific functions and this will be the focus of our future research. Interestingly, the members in JRL-III subfamily have more introns than other subfamilies. It has been reported that intron-rich genes have several advantages for species evolution, including increased gene length and recombination frequency between genes. Additionally, introns often contain regulatory elements such as enhancers, and differential splicing of introns allows a single gene to produce multiple protein isoforms (Back and Walther, 2021). Therefore, the increase of introns in the JRL-III subfamily may facilitate an adaptation to the changing environment.

TD and WGD play indispensable roles during the evolution of gene family (Ohno, 1971; Semple and Wolfe, 1999; Xiang et al., 2011; Zhang et al., 2022). Our results suggest that tandem replication events contribute more to the evolution of Gramineous *JRL* genes (**Figure 4**). The TD is also a process of new gene generation and functional diversification, which helps plants adapt to environmental changes. Therefore, the TD events analysis hints that these JRL proteins might play specific functions in plant environmental adaptation. Purification selection pressure targets to gradually remove harmful and detrimental genetic variants out through evolution, thus beneficial variants may confer advantages (Chang et al., 2015). Ka/Ks values indicates that the evolution of *JRL* genes in *Arabidopsis* and Gramineous may be predominantly influenced by purification selection (**Figure 5**), and this could promote their better adaptation to complex environmental changes. And the extensive involvement of *JRL* genes in both biological and abiotic stresses (**Figures 8**-**10**) further support this hypothesis.

The expression profiles indicated that *OsJRL* and *ZmJRL* genes were expressed in different tissues. Specifically, *OsJRL* genes exhibited high expression levels in the roots, while *ZmJRL* genes were predominantly expressed in inflorescences (**Figure 8**). As sessile organisms, Gramineous crops have evolved the ability to adapt to various unfavorable environments, such as high salt, drought, cold and heat to some extent. And we investigated and found that *OsJRL* genes respond diversely to drought, cold, salt, and heat stresses, which aligns with the reported essential roles of *JRL* genes (Abebe et al., 2005; Ma et al., 2021; Gao et al., 2023). Interestingly, the expression levels of the orthologous genes *OsJRL12/ZmJRL15* are up-regulated under salt stress (**Figure 9**), while the orthologous genes *OsJRL6/ZmJRL16* exhibit opposing expression patterns under salt stress. Though the mechanisms by which *JRL* genes respond to abiotic stresses require further investigation, these results indicate that *JRL* gene is not only functionally differentiated but also functionally conserved across different species. Collectively, these findings illuminate that *JRL* genes can effectively respond to complex and adverse environmental conditions.

## 4 Materials and methods

### 4.1 Plant materials and treatments

The plant material utilized in this study was the rice variety Zhonghua 11 (ZH11). Rice seeds were initially soaked and incubated in purified water overnight in a dark incubator set at 28°C. After two days, uniform seedlings were selected and transferred to be grown in Kimura nutrient solutions, and they were grown under a photoperiod of a 10-hour light/14-hour darkness, with a relative humidity of 50%. Following a 10-day cultivation period, various stress treatments were administered. Drought stress was simulated by application of 30% polyethylene glycol (PEG6000), while salt stress was treated using 150 mM NaCl. Plant roots were collected from both the control and experimental groups at 0, 12, 24, and 48 hours post-treatment. The samples were rapidly frozen in liquid nitrogen, and stored at –80°C for future analysis. The experiment was conducted with three biological replicates.

### 4.2 Sources of genomic data for different species

The species selected for this study include *Oryza sativa (O. sativa), Brachypodium distachyon (B. distachyon), Sorghum bicolor (S. bicolor), Zea mays (Z. mays), Setaria italica (S. italica) and Arabidopsis thaliana (A. thaliana)*. The genome files and gene structure annotation files of the above-mentioned species were downloaded from the Phytozome database (https://phytozome-next.jgi.doe.gov/) (Goodstein et al., 2012). The sequences of the JRL protein family in *A. thaliana* were downloaded from TAIR (https://www.Arabidopsis.org/).

### 4.3 Identification of JRL family proteins in different species

The protein sequences of AtJRLs were used as reference sequences for bidirectional BLAST alignment (E-value < 1e^-5^) to identify potential candidate JRL proteins across various organisms. The unique domain file (PF01419) specific to the JRL protein family was obtained from the Pfam website (http://pfam-legacy.xfam.org/) (Mistry et al., 2021). Subsequently, HMMER 3.0 software was employed to screen for potential candidate JRL family members within the five species under investigation (Potter et al., 2018). The presence of the conserved JRL domain in the candidate proteins was confirmed using NCBI-CDD (https://www.ncbi.nlm.nih.gov/Structure/bwrpsb/bwrpsb.cgi) and SMART (http://smart.embl-heidelberg.de/). Proteins that lacked the JRL domain were excluded from the candidate list based on this analysis.

### 4.4 Evolution analysis of JRL proteins

The JRL protein sequences that we have identified from Gramineae plants were used to construct an evolutionary tree by MEGA7.0 software and neighbor joining (NJ) method (Kumar et al., 2016), with parameter settings of poisson model, pair wise deletion, and 1000 bootstrap replicates.

### 4.5 Analysis of chromosome localization, gene replication, collinearity, and Ka/Ks ratio

The genome location of the *JRL* genes in each species was obtained from their gene structure annotation files (GFF3), named according to their order on chromosomes, and visualized by TBtools. Gene replication events were explored by the Multiple Collinearity Scan Toolkit (MCScan), with parameters set to default (Wang et al., 2012). Using rice as a reference, collinearity analysis was conducted with *A. thaliana*, *B. distachyon*, *S. bicolor*, *Z. mays* and *S. italica*. The results were visualized by TBtools software (Chen et al., 2020). The ratio of Ka/Ks for tandem replication and segmental replication gene pairs were calculated through TBtools software (Chen et al., 2020).

### 4.6 Cluster heat map analysis

Gene expression data from different tissues of rice and maize under abiotic stress were obtained from the Plant Public RNA-seq Database (PPRD) (http://ipf.sustech.edu.cn/pub/plantrna/) (Yu et al., 2022). The gene expression was calculated using FPKM values. To highlight gene expression changes, we use Log_2_ converted values with a multiple change of 1+FPKM (treatment group)/1+FPKM (control group). Heat maps were generated by TBtools software.

### 4.7 RNA isolation and quantitative real-time PCR

The primer 5.0 software was used to design specific primers for *JRL* genes in this study (**Supplementary Table 6**). Total RNA was extracted using the KKFast Plant RNApure Kit (ZOMANBIO, ZP405K-2). The cDNA was synthesized by EX RT kit (gDNA remover) (ZOMANBIO, ZR108-3). The quantitative real-time PCR (RT-qPCR) system program was performed according to the previous research (Duan et al., 2023). The gene expression was analyzed by the 2^−ΔΔCT^ method as used in the previous study (Xue et al., 2024).

## 5 Conclusions

In this study, the *JRL* gene families in five Gramineous plants were comprehensively analyzed, resulting in the identification of 101 *JRL* genes, which were categorized into eight subgroups. The gene structures of Gramineae *JRLs* exhibit considerable variability. Duplication events reveal that TD possibly play a major role in the expansion of the gramineous *JRL* gene family. And the expression profile analysis demonstrates that *ZmJRLs* and *OsJRLs* respond differently to drought, cold, salt and heat stress. These findings illustrate that *JRL* genes may function not only conservatively but also divergently across species. Although the biological functions and regulatory mechanisms of JRL proteins remain unclear, our explorations provide a foundation for further analysis of the biological roles of *JRL* genes in Gramineous crops.

## Data Availability

Gene expression data were obtained from the Plant Public RNA-seq Database (PPRD) (http://ipf.sustech.edu.cn/pub/plantrna/) (Yu et al., 2022).
